# Protective effects of Liuweiwuling tablets on carbon tetrachloride-induced hepatic fibrosis in rats

**DOI:** 10.1186/s12906-018-2276-8

**Published:** 2018-07-09

**Authors:** Huimin Liu, Zhenfang Zhang, Huangwanyin Hu, Congen Zhang, Ming Niu, Ruishen Li, Jiabo Wang, Zhaofang Bai, Xiaohe Xiao

**Affiliations:** 1 Department of Pharmacy, 302 Hospital of People’s Liberation Army, Beijing, People’s Republic of China; 2 Animal Laboratory Center, 302 Hospital of People’s Liberation Army, Beijing, People’s Republic of China; 3 China Military Institute of Chinese Medicine, 302 Hospital of People’s Liberation Army, Beijing, People’s Republic of China

**Keywords:** Traditional Chinese medicine, Liuweiwuling tablets, Hepatic fibrosis, Hepatic stellate cell, TGF-β1, TIMPs

## Abstract

**Background:**

Liuweiwuling tablets (LWWL) are an herbal product that exerts remarkable effects on liver protection and aminotransferase levels, and they have been approved by the Chinese State Food and Drug Administration (CFDA). Clinical studies have found that LWWL can inhibit collagen production and reduce the levels of liver fibrosis markers in the serum. Thus, LWWL is expected to have beneficial effects in the treatment of liver fibrosis. The purpose of this study was to evaluate the pharmacological effects of LWWL.

**Methods:**

Hepatic fibrosis was induced in rats via carbon tetrachloride (CCl_4_) treatment. The rats were treated twice weekly for 8 weeks with either 2 mL·kg^− 1^ body weight of a 50% solution of CCl_4_ in olive oil or olive oil alone by oral gavage. A subset of rats received daily intraperitoneal injections of either colchicine (0.2 mg/kg per day), LWWL (0.4, 1.6, or 6.4 g/kg per day), or vehicle (*N* = 12 for all groups) during weeks 9–12. The rats were sacrificed after 12 weeks. Pathological changes in hepatic tissue were examined using hematoxylin and eosin (H&E) and Sirius Red staining. Immunohistochemistry was performed to observe α-smooth muscle actin (α-SMA) and collagen type I (collagen I) protein expression. Western blotting was also used to detect α-SMA protein expression. Real-time quantitative reverse-transcription polymerase chain reaction (RT-qPCR) was used to detect transforming growth factor-1 (TGF-β1), platelet-derived growth factor (PDGF), tissue inhibitor of metalloproteinase-1 (TIMP1), and tissue inhibitor of metalloproteinase-2 (TIMP2) mRNA expression.

**Results:**

LWWL significantly reversed histological fibrosis and liver injury, reduced the hydroxyproline content in liver tissue, and decreased α-SMA and collagen I expression. LWWL also suppressed hepatic stellate cell (HSC) activation by reducing the expression of the profibrogenic factors TGF-β1 and PDGF. The expression levels of TIMP1 and TIMP2, which regulate extracellular matrix (ECM) degradation, were decreased after CCl_4_ injury in LWWL-treated rats.

**Conclusions:**

These data suggest that LWWL may serve as a promising therapeutic agent to reduce fibrogenesis.

## Background

Liver fibrosis is a wound-healing process that occurs in response to chronic liver injury from a variety of etiologies and eventually progresses to liver cirrhosis following persistent inflammation and fibrogenesis [1, 2]. Characteristic features of liver fibrosis include agglutination in the extracellular matrix (ECM) of the liver, increased collagen fiber content, and decreased ECM degradation. Hepatic parenchymal cell injury is the initiating factor in liver fibrosis. Hepatic stellate cell (HSC) activation, proliferation, and transdifferentiation into myofibroblasts are considered pivotal mechanisms of hepatic fibrosis and of the activation of HSC secretion of ECM, including α-smooth muscle actin (α-SMA) and collagen I [3]. The activated HSCs secrete matrix metalloproteinases (MMPs) and matrix metalloproteinase tissue inhibiting factors (TIMPs) and affect the deposition and degradation of the ECM in the liver. Among the MMPs is the main ECM-degrading enzyme, while TIMPs are specific inhibitors of MMPs. Thus, TIMPs inhibit the expression of TIMP1 and weaken the inhibition of MMP1, which promotes ECM degradation and exerts an anti-liver fibrosis effect [4, 5]. Transforming growth factor-1 (TGF-β1), a highly potent fibrogenic cytokine, simulates both small mothers against decapentaplegic homolog (Smad) and mitogen-activated protein kinase (MAPK) signaling in HSCs, thus stimulating collagen I gene expression [6, 7]. TGF-β signaling also plays an important role in the reduction of MMPs and the increase in TIMPs during liver fibrosis [8]. Platelet-derived growth factor (PDGF), a potent proliferative cytokine in HSCs, stimulates mitogen-activated protein kinase/extracellular signal-regulated kinase (MAPK/ERK) and PI3K/Akt/P70S6K signaling in HSCs. These signaling pathways lead to the activation, migration, proliferation, survival, and contraction of HSCs as well as the promotion of ECM secretion and deposition, thus potentially causing hepatic fibrogenesis [9, 10].

Recent studies of the pathophysiological mechanisms and diagnosis of hepatic fibrosis have made considerable progress. Moreover, possible drug targets against hepatic fibrosis have been identified [11]; however, biological or chemical drugs that target hepatic fibrosis have not yet been approved by the FDA (food and Drug Administration) for clinical applications. Traditional Chinese medicine (TCM) has proven to be effective in the treatment of liver fibrosis due to its unique advantages, which have been demonstrated over many years of clinical validation [12]. LWWL is classified under a new category of TCM drugs approved by the CFDA. LWWL is composed of six herbs: *Schisandrae chinensis fructus*, *Fructus Ligustri Lucidi*, *Forsythiae fructus*, *Curcumae rhizoma*, *Perennial sow thistle*, and *Ganoderma spore*. Clinically, LWWL effectively improves collagenase activity, thus increasing collagen degradation in liver tissue and the serum protein content, reduces the liver tissue collagen content, and effectively reduces liver fibrosis markers in the serum. Chronic liver injury is significantly improved and reversed, which promotes ECM degradation and reabsorption to prevent and delay the occurrence of progressive liver fibrosis [12, 13]. Moreover, long-term colchicine treatment in patients with liver fibrosis showed anti-inflammatory, anti-fibrotic, and immunomodulatory effects as well as the relief of portal hypertension [14, 15]. Therefore, colchicine was used as a positive control. In this study, we evaluated the therapeutic effect of LWWL in the treatment of hepatic fibrosis and investigated the mechanism of its antifibrotic effects.

## Methods

### Experimental animals

Sprague-Dawley rats (male, Grade III, 180 ± 20 g, 8 weeks old) were purchased from the Laboratory Animal Center of the Academy of Military Medical Sciences. All the animals were kept in an environmentally controlled breeding room (20–22 °C, 45–50% humidity). This study protocol strictly adhered to the recommendations of the Guidelines for the Care and Use of Laboratory Animals of 302 Military Hospital (No. IACUC-2015-008).

### Experimental drugs

LWWL was purchased from the Shandong Shibojindu Pharmaceutical Company (batch Nos. 150,709, 150,805, 150,804, and 150,812). Colchicine was purchased from Sigma-Aldrich (USA). Olive oil was purchased from Sinopharm (China). Carbon tetrachloride (CCl_4_) was purchased from Beihua.

### Reagents

Hydroxyproline, alanine transaminase (ALT) and aspartate aminotransferase (AST) testing kits were purchased from Nanjing Jiancheng Co., Ltd., China. PCR primers were purchased from Invitrogen. Maxima SYBR Green qPCR Master Mix (2×) and the First Strand cDNA Synthesis Kit were purchased from Thermo Fisher Scientific, Inc. The bicinchoninic acid (BCA) Protein Quantification Kit was purchased from CWBIO (China). Primary antibodies against α-SMA and collagen I were purchased from Abcam (UK), and that against DAPDH was purchased from Cell Signaling Technology (USA).

### Animal model and protocol

Hepatic fibrosis was induced by CCl_4_ as previously described. Male Sprague-Dawley rats were treated twice weekly for 8 weeks with either 2 mL·kg^− 1^ body weight of a 50% solution of CCl_4_ in olive oil or olive oil alone by oral gavage. A subset of rats received daily intraperitoneal injections of either colchicine (0.2 mg/kg per day), LWWL (0.4, 1.6, or 6.4 g/kg per day), or vehicle (*N* = 12 for all groups) during weeks 9–12. The rats were sacrificed after 12 weeks. After sample collection, the rats were euthanized by cervical dislocation. The rat was placed on the lid of the feeding box, and then, the research grabbed the rat’s tail with their right hand, pulled the tail back slightly, quickly pressed the thumb and forefinger of their left hand down on the head, and dislocated the rat’s neck with both hands. All the animals received humane care in compliance with the Chinese Animal Protection Act and according to National Research Council criteria. The protocol was approved by the Committee on the Ethics of Animal Experiments of the 302 Military Hospital.

### Histological analysis and immunohistochemistry

Tissues were fixed in 4% paraformaldehyde and embedded in paraffin. Sections (5-μm thickness) were prepared for H&E and Sirius Red staining as well as α-SMA (1:500) and collagen I (1:200) immunohistochemistry. All the procedures were performed as previously described.

### Measurement of the hydroxyproline content and serum ALT/AST levels

Hydroxyproline, ALT, and AST kits were utilized in accordance with the manufacturers’ protocols. Briefly, serum samples were obtained by separating the supernatant from the blood. After centrifugation (3000 rpm for 10 min), serum ALT and AST levels and the hydroxyproline content were measured using an Olympus AU5400 Automatic Biochemistry Analyzer (Olympus Optical, Tokyo, Japan).

### RNA isolation and quantitative reverse-transcription polymerase chain reaction (RT-PCR) analysis

The total RNA was isolated from liver tissue using TRIzol reagent (Invitrogen). RNA (1 μg) was reverse-transcribed to complementary DNA (cDNA) using a First Strand cDNA Synthesis Kit (Thermo Fisher Scientific, Inc.). Quantitative RT-PCR was performed to measure Acta2 (encoding α-SMA), collagen I, TGF-β1, PDGF, TIMP1, and TIMP2 mRNA expression using Power SYBR Green PCR Master Mix (Life Technologies, Thermo Fisher Scientific, Inc.) on an ABI Prism 7500 Sequence Detection System. Data analysis was performed using the 2^−ΔΔCT^ method for relative quantification. All the gene expression levels were calculated relative to GADPH. Primer sequences are shown in Table 1.

**Table 1:** Primer Sequences Used in This Study

Target gene	Forward primer (5′- 3′)	Reverse primer (5′- 3′)
Acta2	GAACACGGCATCATCACCAA	CAAGGTCGGATGCTCCTCTG
Collagen I	CTCCTGGCAAGAACGGAGA	CCAGCTGTTCCAGGCAATC
TGF-β1	CCGCAACAACGCAATCTATG	AGTTCTACGTGTTGCTCCACAGT
PDGF	TCTGCTGCTACCTGCGTCTG	AGGCGCTGAAGGTCATCAA
TIMP1	GCCTCTGGCATCCTCTTGTT	CCAGGTCCGAGTTGCAGAA
TIMP2	TATTGTGCCCTGGGACACG	GTCCATCCAGAGGCACTCATC
GAPDH	GCATCCTGCACCACCAACT	GCAGTGATGGCATGGACTGT

### Western blot analysis

Liver tissues were homogenized in lysis buffer (150 mM of NaCl, 1% Nonidet P-40, 0.1% SDS, 50 mM of Tris-HCl, pH = 7.4, 1 mM of EDTA, 1 mM of PMSF, and 1× Roche Complete Mini Protease Inhibitor Cocktail), and protein concentrations were determined using the BCA Protein Assay Kit according to the manufacturer’s protocol. The proteins were separated by gel electrophoresis and transferred to membranes, followed by blocking with 5% *w*/*v* skim milk in 1× TBST for 1 h at room temperature and overnight incubation with primary antibodies against α-SMA (1:200) and GAPDH (1:1000) at 4 °C with gentle shaking. After incubation with the secondary antibody (anti-rabbit IgG) for 1 h, the blot was processed according to the recommended procedure. The gray densities of the protein bands were normalized by using the GAPDH density as an internal control, and the results were further normalized to the control.

### Statistical analysis

All the data are presented as the means ± standard deviation (x ± s) and were analyzed using SPSS 23.0. Differences between experimental groups were analyzed by analysis of variance (ANOVA) followed by post-hoc tests. *P* < 0.01 was regarded as highly statistically significant, and *P* < 0.05 was regarded as statistically significant.

## Results

### LWWL inhibited CCl_4_-induced liver fibrosis in rats

**Fig. 1:**
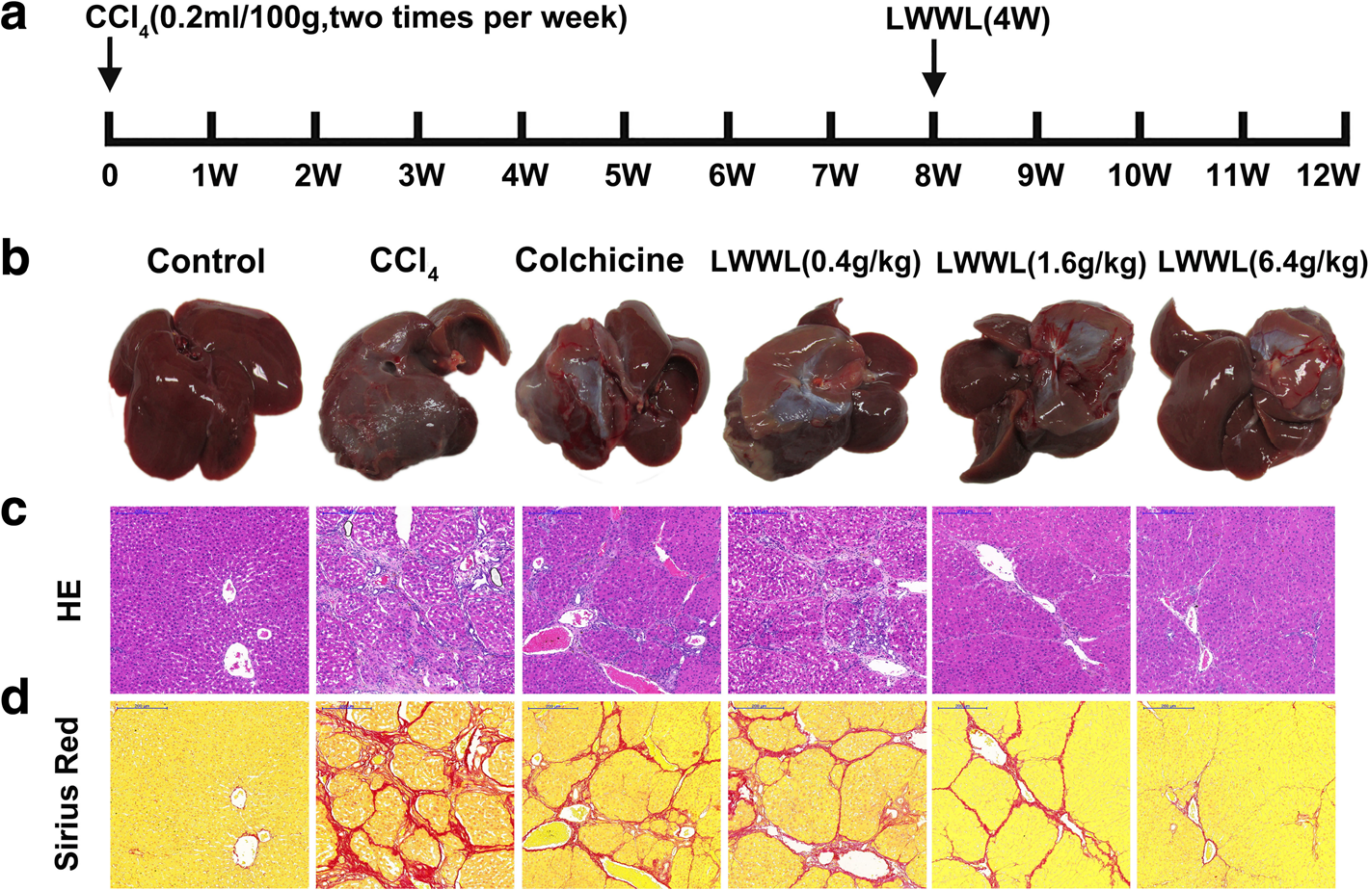
LWWL inhibited CCl_4_-induced rat liver fibrosis. **a** Schematic representation of the CCl_4_-induced hepatic fibrosis in rats; **b** Representative rat livers at the time of sacrifice; **c** Representative rat livers stained with HE at the time of sacrifice; **d** Representative rat livers stained with Sirius Red (× 100) at the time of sacrifice

The effects of LWWL on fibrogenesis were tested using the well-characterized CCl_4_ rat model. The rats that were injured with CCl_4_ by oral gavage reliably developed liver fibrosis after 8 weeks. The experimental design is shown in Fig. 1a. Liver pathological observation is the gold standard for the clinical diagnosis of liver fibrosis. Compared with the control group, the liver tissues of the model group showed severe adhesion of liver tissue, and the texture was significantly stiffened, the surface roughened, and the luster was lost, forming a lumpy mass. After treatment with LWWL (0.4, 1.6, or 6.4 g/kg) dramatically inhibited CCl_4_-induced hepatic fibrogenesis, the adhesion of liver tissues decreased, and the texture became soft and lustrous, as indicated by the striking morphological alteration (Fig. 1b). H&E and Sirius Red staining results showed that compared with the control group, the livers of the model group had a remarkable ECM area. Furthermore, the groups treated with LWWL showed markedly reduced collagen levels in H&E and Sirius Red staining sections (Fig. 1c, d).

### LWWL attenuated liver function and the hydroxyproline content in rats

**Fig. 2:**
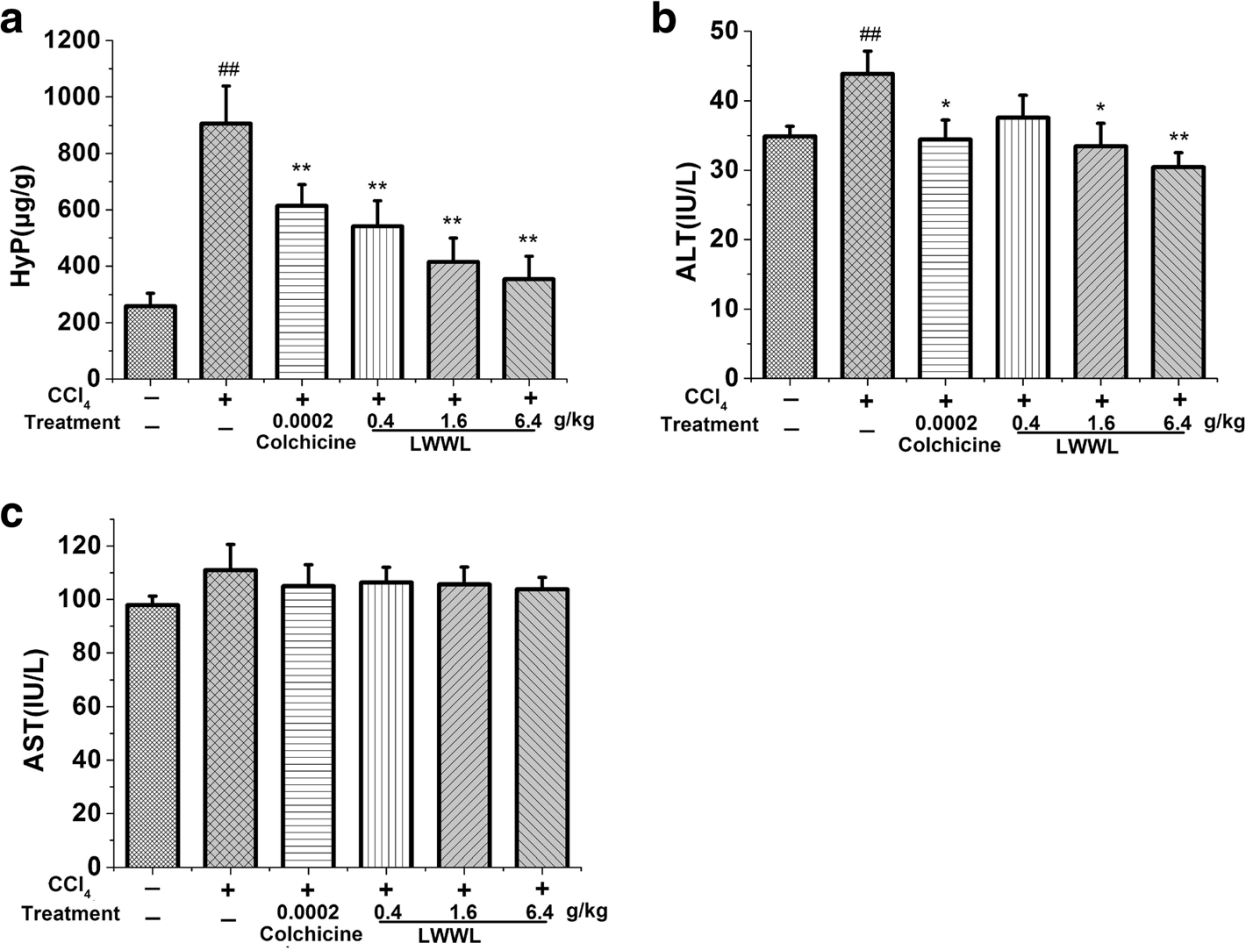
LWWL attenuated liver function and the hydroxyproline content in rats. **a** Effect of LWWL on hydroxyproline levels in liver tissue; **b** Effect of LWWL on serum ALT levels; **c** Effect of LWWL on serum AST levels. ##*P* < 0.01 vs. normal group; *P < 0.05, **P < 0.01 vs. model group

Liver function tests were used to assess injury. Hydroxyproline, an indicator of collagen deposition. CCl_4_ significantly increased the levels of the serum ALT and hydroxyproline in liver tissue compared with those of the control group, and the effects of LWWL revealed decreased ALT levels but no changes in serum AST levels. Meanwhile, LWWL also reduced the level of hydroxyproline, which directly reflects the amount of liver fibrosis (Fig. 2a, b, c).

### LWWL inhibited HSC activation in CCl_4_-induced hepatic fibrosis in rats

**Fig. 3:**
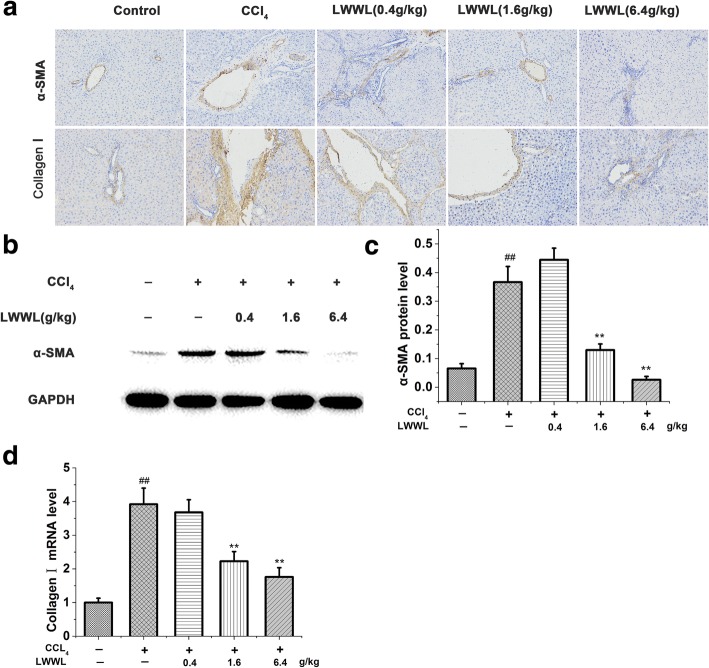
LWWL inhibited HSCs activation in CCl_4_-induced hepatic fibrosis rats. **a** Immunohistochemical analysis of α-SMA and collagen I (× 200); **b** Western blot analysis of α-SMA; **c** Densitometry of α-SMA expression levels; **d** RT-PCR for collagen I. ##*P* < 0.01 vs. normal group; **P < 0.01 vs. model group

HSCs have been recognized as the main matrix-producing cells during the process of liver fibrosis. α-SMA is a commonly used marker for the activated HSCs [16]. Collagen I is the prototype constituent of the fibril-forming matrix in fibrotic liver, and its expression is regulated by transcriptionally as described in several reviews [17, 18]. In this animal model, LWWL reduced liver injury, which is one potential mechanism by which fibrosis progression was inhibited. CCl_4_ injury markedly increased the α-SMA and collagen I mRNA and protein levels. In contrast, LWWL treatment dramatically suppressed the messenger RNA (mRNA) expression of markers for HSC activation, such as α-SMA and collagen I, in a dose-dependent manner in CCl_4_-injured fibrotic livers (Fig. 3a, c, d). In line with the former, the protein expression of α-SMA was significantly inhibited in the LWWL-treated rat livers (Fig. 3b).

### LWWL inhibited the progression of profibrogenic factors and collagen deposition in CCl_4_-induced hepatic fibrosis in rats

**Fig. 4:**
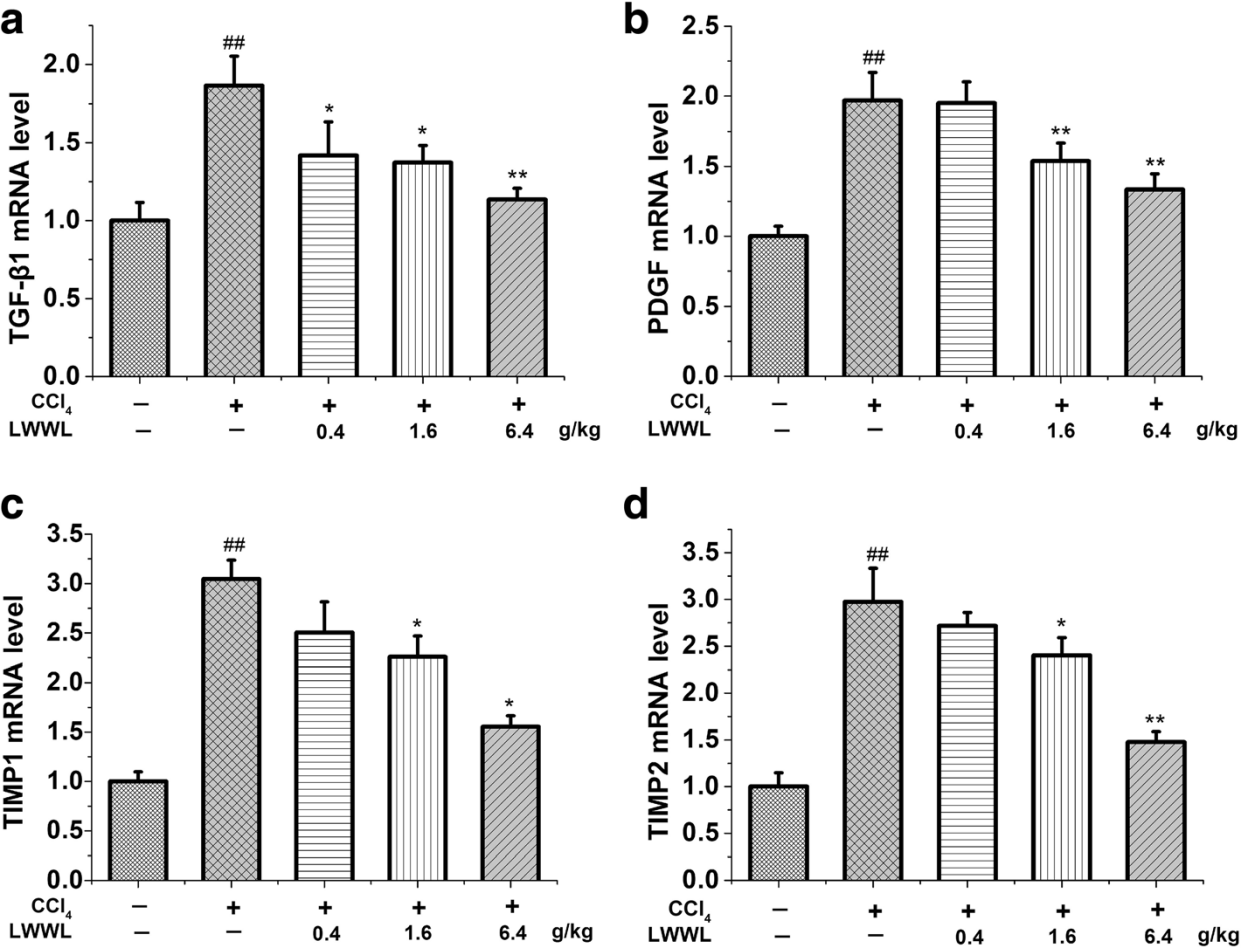
LWWL inhibited the progression of profibrogenic factors and deposition of collagen in rats with CCl_4_-induced hepatic fibrosis. **a** RT-PCR for TGF-β1; **b** RT-PCR for PDGF; **c** RT-PCR for TIMP1; **d** RT-PCR for TIMP2. ##*P* < 0.01 vs. normal group; *P < 0.05, **P < 0.01 vs. model group

Several profibrogenic factors (e.g., TGF-β1 and PDGF) that bind to their cognate receptors on the HSC surface activate the corresponding signaling pathway, as well as the proliferation of HSCs, excessive ECM deposition, and hepatic fibrogenesis [19, 20]. The proliferation and activation of HSCs is critical during the process of liver fibrosis, and TGF-β1 and PDGF are the most effective factors that promote HSC proliferation and liver fibrosis. We observed that TGF-β1 expression normally increases over time in CCl_4_-injured rats. We also observed a consistent increase in the expression of PDGF but a decrease in LWWL-treated livers (Fig. 4a, b). TIMPs are an important family of enzymes that regulate the activity of MMPs. TIMPs can inhibit the activity of MMPs and reduce ECM degradation, leading to liver fibrosis. TIMP-1 and TIMP-2 are mainly produced by HSCs and are upregulated in various human liver diseases [21]. TIMP-1 production can be attributed to activated HSCs [22]. Thus, TIMP-1 is a central molecule in liver fibrosis. In cultured liver cells, TIMP-2 mRNA expression has not been detected in native hepatocytes, but it has been detected in activated HSCs, Kupffer cells, and rat liver myofibroblasts [23]. In fibrogenesis, TIMP-2 mRNA expression is restricted to the early stages, during which a transient increase in TIMP-2 activates MMP-2, followed by the pericellular degradation of the normal liver matrix [24]. In this study, we assessed the TIMP1 and TIMP2 mRNA expression in CCl_4_-injured livers, which was increased compared with that in the control. However, treatment with LWWL decreased TIMP mRNA levels in a dose-dependent manner in CCl_4_-injured rats (Fig. 4c, d).

## Discussion

Hepatic fibrosis results in severe hepatic injury following ECM accumulation. This phenomenon causes many chronic liver diseases, including chronic HCV and HBV infections, chronic alcoholic liver disease, and nonalcoholic steatohepatitis. These occurrences can lead to cirrhosis or hepatocellular carcinoma (HCC). Thus, preventing hepatic fibrosis represents an important strategy for the treatment of chronic liver disease. A variety of small molecules and biological agents are being developed to treat hepatic fibrosis [20]. However, several pathogenic mechanisms mediate hepatic fibrosis, and these agents have not yet been successfully employed in anti-hepatic fibrosis treatment regimens in a clinical setting. Many clinical and experimental studies have shown that TCMs are advantageous because they exert pharmacological effects by acting on multiple targets during the treatment of complex diseases [25, 26], which can delay or curb liver fibrosis and even cirrhosis. Their mechanism of action includes liver injury prevention, inhibition of ECM synthesis, activation of HSCs, and ECM degradation. Thus, TCMs with anti-liver fibrosis properties are becoming the focus of studies on the prevention and treatment of this disease [27–30].

LWWL, a Chinese medicinal formula, has historically been used to reduce ALT levels in patients with chronic hepatitis. Moreover, LWWL-specific adverse drug reactions have not yet been observed in a clinical setting. The present study investigated the herbal prescription LWWL, which exerts protective effects on the liver by reducing serum ALT levels. In the pharmacodynamic evaluation section of this study, LWWL did not significantly interfere with the level of AST in the serum of rats with CCl_4_-induced hepatic fibrosis. The above results were repeated at least two times, and the error of detection could be eliminated as a cause. Some studies on anti-hepatic fibrosis exist, but only ALT data were available, and AST data did not appear [31, 32]. A possible reason for the above results is that the model is a classic model of hepatic fibrosis, but the modeling time is as long as 8 weeks. Therefore, it may be that long-term stimulation by CCl_4_ is not sensitive to changes in AST. LWWL inhibited HSC activation associated with a reduction in the mRNA and protein levels of α-SMA. LWWL administration also markedly suppressed fibrotic pathological changes and elevated the hydroxyproline content in parallel with a reduction in the mRNA levels of collagen I in the liver treated with CCl_4_. In fact, we gathered the protein expression of collagen I several times before; during the experiment, we changed to different antibodies, and the experimental conditions were constantly improved, but the band was blurred. Thus, the result was very difficult to determine, and the final data are not displayed. Because there is currently no reliable anti-hepatic fibrosis drug, it is difficult to determine which drug concentrations should be utilized for antifibrotic experiments. Upon designing this experiment, we decided to use colchicine as the positive control drug, but taking into account that the mechanism may be different from that of LWWL, it did not appear in the later data.

Activated HSCs proliferate and migrate to injured sites, secreting large amounts of ECM, which alters the normal architecture of the liver and initiates several positive feedback pathways that lead to liver fibrosis [33, 34]. Therefore, strategies to eliminate or normalize activated HSCs are critical for liver fibrosis therapy. Fibrotic pathologies are associated with increased levels of TGF-β1 that initially recruit inflammatory cells and fibroblasts into an area of injury and then stimulate these cells to produce cytokines and ECM [35]. TGF-β directly increases the synthesis of ECM components such as Collagen I. In addition, a significant increase in the TGF-β expression is observed in the activated HSCs and MFBs, thus indicating that TGF-β acts as an autocrine positive regulator for ECM production [36]. In the present study, LWWL markedly decreased the expression of TGF-β1 and PDGF in the livers of experimental rats after treatment with CCl_4_. TGF-β inhibits the expression of tissue collagenase, which is a specific enzyme for degradation of mammalian intestinal collagens, and enhances the production of inhibitors of such ECM-degrading enzymes as TIMPs [37]. In this respect, TIMPs are the main targets of the TGF-β signaling pathway [38, 39]. LWWL caused significant reductions in TIMP-1 and TIMP-2 mRNA levels in CCl_4_-treated rats. Thus, decreasing TIMP-1 or TIMP-2 levels may lead to an increase in ECM degradation. In the present study, TGF-β1 mRNA expression was significantly inhibited by LWWL, which may be one mechanism by which TIMP expression is regulated. Because of their importance in fibrosis, TIMPs may represent an attractive therapeutic target. These results suggest that LWWL prevents hepatic fibrosis, at least in part, by negatively regulation of TGF-β signaling pathway to inhibit the activation and proliferation of HSCs. Salidroside is a phenolic glycoside and possesses various pharmacological properties which can isolated from *Fructus Ligustri Lucidi* in the herbal prescription LWWL. It’s reported that treatment with salidroside can protects against bleomycin-induced pulmonary fibrosis via inhibition of NF-κB and TGF-β1/Smad-2/− 3 pathways [40]. Therefore, understanding the regulatory mechanisms of the TGF-β signal between physiologic and pathologic situations will be essential in the design of new therapeutic approaches for various diseases caused by a deregulation of the TGF-β signal. Thus, antagonists of the TGF-β signal could be applied in liver fibrosis. If the active compounds are available to the public, our present knowledge base of the physiologic regulation in activated HSCs and pathologic deregulation in MFBs of autocrine TGF-β signal will help elucidate the clinical application of such drugs for liver fibrosis in the future.

## Conclusions

In summary, we used the well-characterized CCl_4_ rat model to examine the antifibrotic effects of LWWL. In the present study, LWWL attenuated CCl_4_-induced liver fibrosis and improved liver function. The antifibrotic effects of LWWL were associated with its reduction of collagen synthesis, suppression of HSC activation, and promotion of ECM degradation. Taken together, the results of this study provide new opportunities for the use of LWWL in the treatment of liver fibrosis. However, the specific mechanism involved still needs to be determined.

## Abbreviations

ALT: alanine transaminase
ANOVA: analysis of variance
AST: aspartate aminotransferase
BCA: bicinchoninic acid
CCl_4_: carbon tetrachloride
cDNA: complementary DNA
CFDA: Chinese State Food and Drug Administration
collagen I: collagen type I
ECM: extracellular matrix
ELISA: enzyme-linked immunosorbent assay
FDA: food and Drug Administration
HE: hematoxylin and eosin
LWWL: Liuweiwuling tablets
MAPK/ERK: mitogen-activated protein kinase/extracellular signal-regulated kinase
MFB: myofibroblasts
MMPs: matrix metalloproteinases
PDGF: platelet-derived growth factor
RT-qPCR: real-time quantitative reverse-transcription polymerase chain reaction
TCM: traditional Chinese medicine
TGF-β1: transforming growth factor β1
TIMPs: tissue inhibitor of metalloproteinases
VEGF: vascular endothelial growth factor
α-SMA: α-smooth muscle actin
